# Whole Genome Sequence of an Edible Mushroom *Stropharia rugosoannulata* (Daqiugaigu)

**DOI:** 10.3390/jof8020099

**Published:** 2022-01-20

**Authors:** Shuwen Li, Shuxue Zhao, Chunhui Hu, Chengzhi Mao, Lizhong Guo, Hailong Yu, Hao Yu

**Affiliations:** 1 Shandong Provincial Key Laboratory of Applied Mycology, School of Life Sciences, Qingdao Agricultural University, 700 Changcheng Road, Qingdao 266109, China; swli@qau.edu.cn (S.L.); 11201011028@stu.ouc.edu.cn (S.Z.); 201201012@qau.edu.cn (C.H.); 20212106012@stu.qau.edu.cn (C.M.); 198701007@qau.edu.cn (L.G.); 2 National Engineering Research Center of Edible Fungi, Institute of Edible Fungi, Shanghai Academy of Agricultural Sciences, Shanghai 201403, China

**Keywords:** genome, *Stropharia rugosoannulata*, mushroom, lignocellulose degradation, CAZymes

## Abstract

*Stropharia rugosoannulata,* also known as Daqiugaigu in China, is a well-known edible mushroom that has been widely cultivated in China in recent years. Many studies have focused on its nutrients, bioactive compounds, and lignin degradation capacity, although there are few molecular and genetic breeding studies due to the lack of genomic information. Here, we present the 47.9 Mb genome sequence of an *S. rugosoannulata* monokaryotic strain (A15), which has 20 contigs and an N50 of 3.64 Mb, which was obtained by a combination of Illumina and Nanopore sequencing platforms. Further analysis predicted 12,752 protein-coding genes, including 486 CAZyme-encoding genes. Phylogenetic analysis revealed a close evolutionary relationship between *S. rugosoannulata* and *Hypholoma sublateritium*, *Psilocybe cyanescens*, and *Galerina marginata* based on single-copy orthologous genes. Proteomic analysis revealed different protein expression profiles between the cap and the stipe of the *S. rugosoannulata* fruiting body. The proteins of the stipe associated with carbon metabolism, energy production, and stress-response-related biological processes had higher abundance, whereas proteins involved in fatty acid synthesis and mRNA splicing showed higher expression in the cap than in the stipe. The genome of *S. rugosoannulata* will provide valuable genetic resources not only for comparative genomic analyses and evolutionary studies among *Basidiomycetes* but also for alleviating the bottlenecks that restrict the molecular breeding of this edible mushroom.

## 1. Introduction

*Stropharia rugosoannulata* is a widely cultivated edible mushroom in China and northern temperate zones throughout the world. *S. rugosoannulata* has a pleasant flavor, is rich in nutrients, and is a good source of proteins, mineral elements [1,2,3], and bioactive compounds, including polysaccharides [4,5,6,7,8,9], steroids [10,11,12], lectins [13], ceramides [14], and phenols [15], which possess antioxidant properties [4,16], endoplasmic reticulum stress-lowering capabilities [12], and hemagglutinin [13] and hypoglycemic activities [6]. In contrast to most wood rot edible mushrooms, *S. rugosoannulata* is mainly cultivated with straw and cornstalk. Therefore, its cultivation can reduce the consumption of forest resources. Furthermore, *S. rugosoannulata* shows high resistance to pathogens, such as filamentous fungi and nematodes [17], which may be due to the existence of acanthocytes [18] or chemotaxis compounds [19]. Therefore, it can be inoculated with unsterilized media, which reduce the consumption of energy. Because of its robustness and good adaptability, *S. rugosoannulata* can be cultivated in different environments and is recommended as a good food source for developing countries by the Food and Agriculture Organization.

Despite its nutrients and bioactive compounds, several studies focused on the development, lignocellulose degradation capacity, and breeding of *S. rugosoannulata*. *S. rugosoannulata* is a good degrader of environmental pollutants, and it can produce a variety of laccases and peroxidases, which can break down polycyclic aromatic hydrocarbons, dyes, and pharmaceutically active compounds, as well as dissipate quintozene [20,21,22]. Yan et al. isolated the monokaryotic strains of *S. rugosoannulata*, which were differentiated easily using the randomly amplified polymorphic DNA (RAPD) technique [23,24]. By studying their mating system, the authors demonstrated that the mating type of *S. rugosoannulata* was heterothallic and tetrapolar. In a follow-up study, Yan et al. optimized the protoplast preparation method of *S. rugosoannulata*, which is useful in crossbreeding or transformation [25]. However, compared with other mushrooms, such as *Auricularia*, *Lentinuta*, *Agaricus*, *Coprinopsis,* and *Fillumina* [26,27,28,29,30,31,32,33,34], there are few biological and genetic studies on *S. rugosoannulata*, which impedes the breeding of high-quality cultivars. The lack of genomic information is one of the main reasons limiting the further study of this mushroom.

In the present study, we used Illumina and Nanopore platforms to sequence the *S. rugosoannulata* genome, and we compared its genome, evolutionary relationships, and lignocellulose degradation capacity to other mushrooms. The protein expression profiles of the *S. rugosoannulata* fruiting body were also examined. The *S. rugosoannulata* genome sequence will be helpful for understanding the molecular mechanisms and evolution of this important edible mushroom.

## 2. Materials and Methods

### 2.1. Strains and Culture Condition

The *S. rugosoannulata* GL0170 strain was a gift from the Shandong Provincial Key Laboratory of Applied Mycology. The monokaryotic A15 strain was isolated from the spores of the cultivated GL0170 strain. The monokaryotic strain was confirmed by the absence of a clamp connection and a lower growth rate compared with the dikaryotic mycelia. *S. rugosoannulata* mycelia were cultivated and maintained on potato dextrose agar (PDA) plates as previously described [35].

### 2.2. Genome Sequencing

The *S. rugosoannulata* A15 strain was inoculated on PDA plates that were covered with cellophane, and 2.0 g of mycelia was scraped and transferred into a centrifuge tube for DNA and RNA extraction. The genome was extracted using the modified CTAB method [31]. The genomic DNA concentration was determined using the Nanodrop spectrophotometer (Thermo Fisher Scientific, Waltham, MA, USA), and the purity and integrity were determined using the Agilent 2100 bioanalyzer (Agilent Technologies, Santa Clara, CA, USA). The *S. rugosoannulata* genome and RNA-Seq was sequenced using Illumina Navaseq 6000 (paired-end, 2 × 150 bp) and Nanopore PromethlON 48 sequencing technology platforms, with a sequencing depth of >50 for the Illumina platform and >100 for the Nanopore platform. Total RNA was extracted using TRIzol reagent (Takara, Dalian, China) as previously described [35]. RNA-seq was performed using the Illumina platform.

### 2.3. Genome Assembling

The Nanopore reads were assembled using NECAT tools [36], and the assembled results were verified using Pilon software [37] with Illumina fastq data. The integrity of the fungal genome assembly was evaluated using QUAST v5.1.0 with Illumina or Nanopore reads [38].

### 2.4. Gene Prediction and Annotation

Gene predictions were generated from different sources, including *ab initio*-based methods, RNA-seq-based methods, and homology-based methods (Supplementary Table S1). Gene prediction results were integrated using EVM software (Supplementary Table S1). The functional annotations of the predicted protein coding sequences (CDSs) were obtained based on NCBI Nr (September 2021), KEGG (December 2020) [39], GO (June 2021), Pfam (October 2021), Swiss-Prot (September 2021), and CAZymes databases (October 2021). Gene Ontology (GO) terms were predicted using the Blast2GO tool. Protein sequence alignment against the Pfam database [40] was performed using the HMMER tool [41]. The HMMER tool (e-value < 1 × 10^−15^, coverage > 0.35) was also used to identify carbohydrate enzyme genes based on the carbohydrate-related enzyme database dbCAN2 (https://bcb.unl.edu/dbCAN2/blast.php, accessed on 1 October 2021) [42,43].

### 2.5. Collinearity Analysis, Gene Family Construction, and Species Tree Construction

Collinearity analysis was performed using tBTools software [44], based on location information from the GFF3 files of *S. rugosoannulata*, *Coprinopsis cinerea,* and *Agaricus bisporus*. The protein sequences of *S. rugosoannulata*, 14 *Basidiomycetes,* and two *Ascomycetes* were used to calculate the pairwise similarities using the Diamond tool. Gene families were constructed using OrthoFinder v2.5.4 software [45]. Single-copy orthologous genes were extracted for each species and connected for species-scale phylogenetic analysis by multiple sequence alignment using OrthoFinder software. The species tree was visualized using FastTree software.

### 2.6. Protein Extraction and Peptide Digestion

For proteome analysis, stipe and cap tissues were collected from the *S. rugosoannulata* fruiting body (Supplementary Figure S1). Proteins were extracted according to the method reported by Isaacson et al. [46]. Briefly, 1 g fruiting body tissue was added into a 2 mL centrifugation tube with two 5-mm stainless steel beads. The tissue was lysed with JXFSTPRP-24 TissueLyser (Jingxin, Shanghai, China) at 60 Hz for 120 s and 2 mL of PheExtract (0.7 M sucrose; 0.1 M KCl; 0.5 M Tris-HCl, pH 7.5, 30 mM DTT, and 50 mM EDTA) was added into the lysed tissue. After vortex for 5 min, 2 mL Tris-saturated phenol (pH = 7.8) was added into the centrifugation tube and vortexed for 5 min. The tube was centrifugated at 15,000× *g*, 4 °C for 10 min, and the upper phenolic phase was transferred to a new tube. An equal volume of PheExtract was added into the tube, and the protein extraction was repeated once. To precipitate the protein, 5 volumes of cold 0.1 M ammonium acetate in methanol were added into the collected phenol phase, and the protein was precipitated overnight at 4 °C. The precipitated protein was collected by centrifugation and washed with cold methanol two times and cold acetone two times. The dried protein was then dissolved in UDT buffer (8 M urea, 10 mM DTT, 100 mM Tris-HCl, pH 8.0). Peptide digestion was performed according to the method reported by Wiśniewski et al. [47]. Two biological replicates were prepared for each group.

### 2.7. Proteomic Analysis and Peptide Searching

LC-MS/MS analysis of peptides was performed using the Nano-LC system, coupled with the Orbitrap Fusion Tribrid mass spectrometer (Thermo Fisher Scientific), as previously described [35]. Peptide identification and quantitation were performed using MaxQuant software [48]. The data were searched against the protein database of *S. rugosoannulata*. Protein abundance estimations were based on intensity-based absolute quantification (iBAQ). MaxQuant output tables were processed using Perseus software [49]. Proteins with missing values were discarded. Data were normalized through dividing by the median value. Proteins with different abundances were selected by the two-sided *t* test with a Benjamini–Hochberg-based FDR cut-off value of 0.05. Data were visualized using the R package.

## 3. Results and Discussion

### 3.1. Genome Assembly of S. rugosoannulata

**Figure 1:**
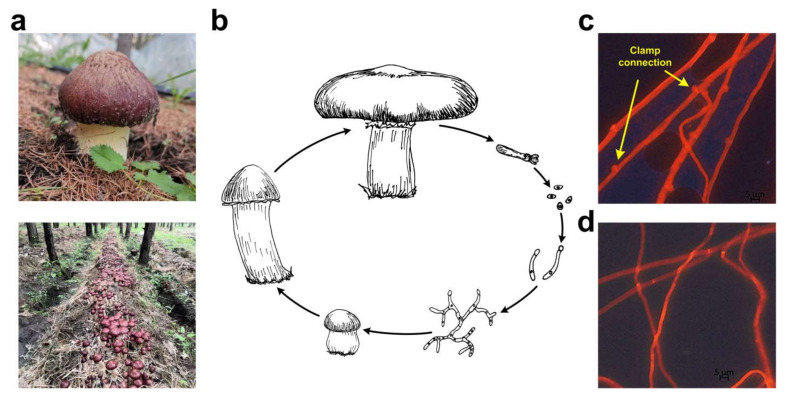
Life cycle of *S. rugosoannulata*. (**a**) Cultivated *S. rugosoannulata* on a forest farm under the crown in China; (**b**) Life cycle of *S. rugosoannulata*, representing the heterothallic and tetrapolar mating system of this mushroom; (**c**) Microscopic observation of dikaryotic mycelia of the GL0170 strain and clamp connections were indicated by arrows; (**d**) Monokaryotic mycelia of the A15 strain.

*S. rugosoannulata* is a popular straw-rotted edible mushroom that has been widely cultivated in China in recent years. The GL0170 strain used in this study was cultivated on Huamugou tree farm, Chifeng, Inner Mongolia, China, from 2018 to 2020 (Figure 1a). *S. rugosoannulata* is a tetrapolar heterothallic fungus (Figure 1b). Clamp connections were observed from dikaryotic mycelia (Figure 1c). The monokaryotic A15 strain was isolated from the basidiospores of the GL0170 strain, and no clamp connection was observed on monokaryotic mycelia (Figure 1d). In addition, the diameter of dikaryotic mycelia was larger than that of monokaryotic mycelia, and the growth rate of dikaryotic mycelia was also faster than that of monokaryotic mycelia (Figure 1).

The genome of the *S. rugosoannulata* A15 strain was sequenced using Nanopore and Illumina sequencing platforms. The raw data were de novo assembled into 20 contigs with an N50 of 3.64 Mbp and an N90 of 2.44 Mbp. The total sequence length was 47.89 Mbp (Table 1). The integrity of the genome (reads mapped ratio) was evaluated using QUAST v5.1.0 software and determined to be 99%, indicating that the assembly of the *S. rugosoannulata* genome was of high quality. The *S. rugosoannulata* strain MG69 was previously sequenced using Illumina technology (QLPO00000000.1) [50] with 17,452 contigs. The genome size of the *S. rugosoannulata* MG69 strain was 50.41 Mbp and slightly larger than that of the A15 strain (Supplementary Table S2) [50], which may have been due to the fact that the MG69 strain is heterokaryotic.

### 3.2. Gene Prediction and Genome Comparisons

A total of 12,752 CDSs were predicted in the *S. rugosoannulata* genome (Table 2), among which there were 12,033 homology-predicted CDSs and transcripts (94.36%) (Supplementary Figure S2), indicating that the prediction results had high reliability. Further proteomic analysis confirmed the high reliability of the CDS predictions. The total length of the encoded genes was 26.99 Mbp, accounting for 56.35% of the whole genome. The average length of each gene was 2116.71 bp. The average exon and intron numbers were 6.55 and 5.55, respectively.

**Figure 2:**
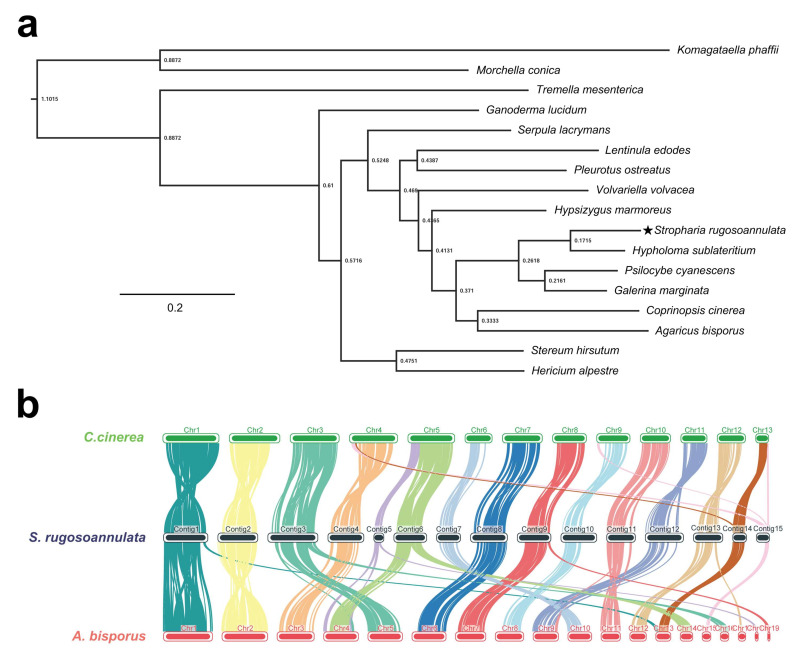
Comparison of genomes between *S. rugosoannulata* (indicated by star) and 16 other fungi (14 *Basidiomycetes* and 2 *Ascomycetes*). (**a**) Phylogenetic tree of 17 fungi based on single-copy orthologous genes. *Komagataella phaffii* and *Morchella conica* served as the outgroups. Tree scale = 0.2; (**b**) The genome collinearity among *S. rugosoannulata*, *A. bisporus*, and *C. cinerea*.

In cluster analysis of 16 fungal species, 7766 (46.6%) of 16,680 groups containing at least one *S. rugosoannulata* gene were identified (Table 3). There were 197 species-specific orthogroups containing 881 species-specific genes in *S. rugosoannulata* (Table 3). A total of 647 single-copy orthologous genes in 17 fungi were used to construct the phylogenetic tree. As shown in Figure 2a, *S. rugosoannulata* was classified into one group with *Hypholoma sublateritium* and was clustered into one group with *Psilocybe cyanescens* and *Galerina marginata* by ortholog-based clustering analysis. The four fungi belonged to one family, *Strophariaceae*, which is consistent with the taxonomy based on the morphological traits in NCBI taxonomy.

The genomes of *A. bisporus* and *C. cinerea* were assembled at the chromosome level, and both had a close genetic relationship with *S. rugosoannulata* based on phylogenetic tree analysis. Therefore, chromosome collinearity analysis of these three species was performed using tBTools (Figure 2b). Several translocations occurred between these species, mainly in subtelomeric regions. In Chr14 and Chr15 of *S. rugosoannulata*, major chromosomal rupture and fusion events occurred between *S. rugosoannulata* and *C. cinerea*. In addition, Chr5 and Chr6 of *S. rugosoannulata* may belong to the same chromosome. More rupture and fusion events occurred between *S. rugosoannulata* and *A. bisporus* than those between *S. rugosoannulata* and *C. cinerea*. Rupture and fusion events were identified in Chr1, Chr3, Chr5, Chr6, Chr9, Chr11, and Chr13 of *S. rugosoannulata* compared to chromosomes from *A. bisporus*. Collinearity analysis indicated that *S. rugosoannulata* had a close genetic relationship with *C. cinerea*. Five chromosomes (Chr16 to Chr20, 444 kb, 0.9% of the total genome) showed no similarity with the genome of the other two strains. These results demonstrated the high quality of the *S. rugosoannulata* genome assembly.

### 3.3. Annotation of the S. rugosoannulata Genome

The predicted gene sequences were annotated functionally by sequence alignment against sequences in Nr, Swiss-Prot, and Pfam databases. Most genes were matched using the Nr (11,599 genes) database, followed by Pfam (7672 genes) and Swiss-Prot (6193 genes) databases (Supplementary Table S3).

**Figure 3:**
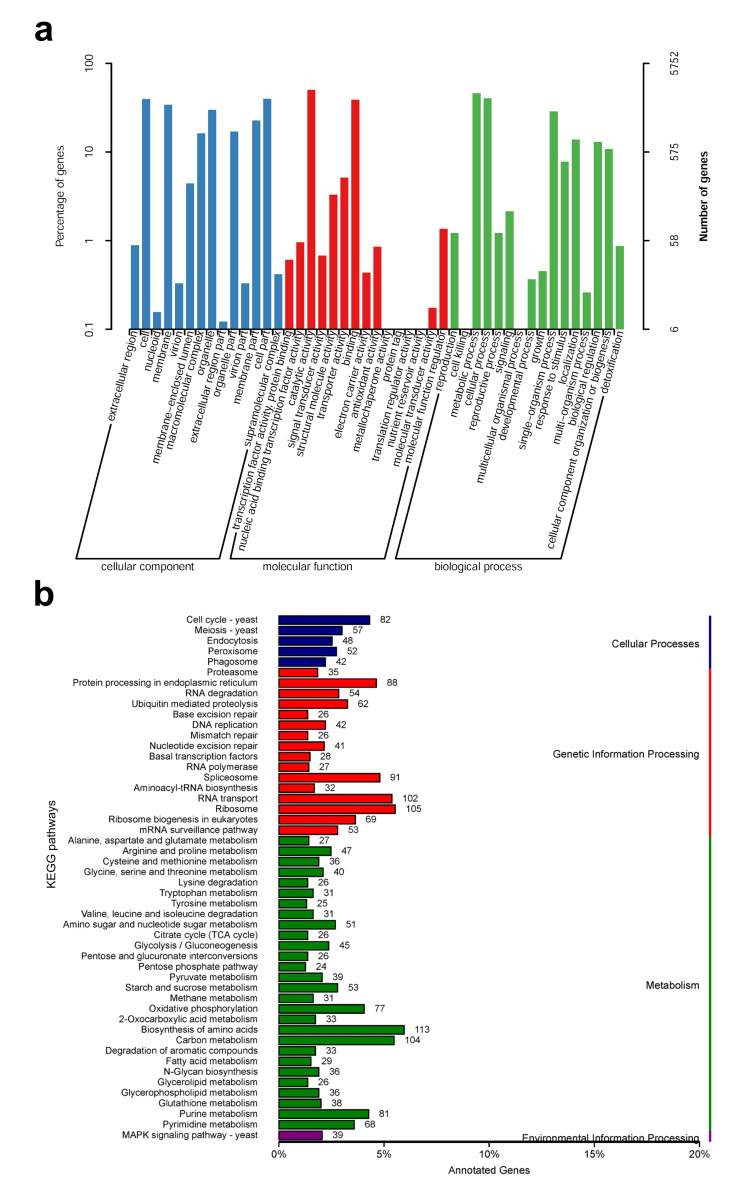
Classification statistics of GO and KEGG annotations of *S. rugosoannulata* genome. (**a**) The GO function annotation of *S. rugosoannulata*; (**b**) The KEGG pathway annotation of *S. rugosoannulata*.

According to the GO database, 5752 predicted proteins accounting for 45.1% of the entire genome were distributed into three functional categories, namely, cellular components, molecular functions, and biological processes. The cellular components covered were mainly distributed across cell organelles and membranes. The molecular function component was mainly distributed across catalytic activity, binding, and transporter activity. For biological processes, the metabolic processes, cellular processes, and single-organism processes contained the most proteins (Figure 3a). To further understand the functions of *S. rugosoannulata* proteins, 3330 (26.1%) proteins were assigned to their orthologs in the KEGG database. A total of 108 pathways were identified, which were classified as those related to cellular processes, genetic information processes, metabolic processes, and environmental information processes (Figure 3b). The number of genes in the biosynthetic amino acid pathway was the highest. For genetic information processes, the ribosome pathway was the most involved, whereas, for cellular processes, the cell cycle–yeast pathway was the most involved. For environmental information processes, only the MAPK signaling-yeast pathway was predicted (Figure 3b).

### 3.4. Carbohydrate Active Enzymes (CAZymes)

**Figure 4:**
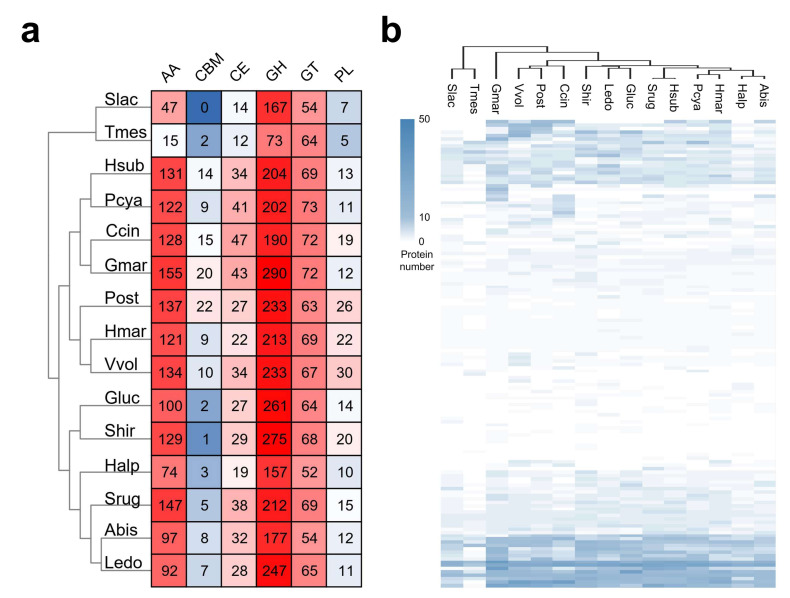
CAZymes in *S. rugosoannulata* and other fungi. (**a**) Heatmap representing CAZyme categories distributed in *S. rugosoannulata* and 14 other fungi. The color indicated the large (red) and small (blue) numbers of the proteins in each category.; (**b**) Heatmap representing CAZyme families distributed in *S. rugosoannulata* and 14 other fungi. GH, glycoside hydrolase; GT, glycosyltransferase; PL, polysaccharide lyase; CE, carbohydrate esterase; CBM, carbohydrate-binding module; AA, auxiliary activity; Abis, *A. bisporus* var. burnettii JB137-S8; Ccin, *C. cinerea* okayama7#130; Gluc, *Ganoderma lucidum* G.260125-1; Gmar, *G. marginata* CBS 339.88; Halp, *Hericium alpestre* DSM 108284; Hmar, *H. marmoreus* 51987-8; Hsub, *H. sublateritium* FD-334 SS-4; Ledo, *Lentinula edodes* NBRC 111202; Pcya, *P. cyanescens* 2631; Post, *Pleurotus ostreatus* PC15; Shir, *Stereum hirsutum* FP-91666 SS1; Slac, *Serpula lacrymans*; Srug, *S. rugosoannulata* A15; Tmes, *Tremella mesenterica* ATCC 28783; Vvol, *Volvariella volvacea*. The color indicated the numbers of the proteins in each category.

CAZymes are one of the most important gene families in the fungal genome, and they are not only involved in lignocellulose degradation but also in many other biological processes, such as development and the stress response [51,52]. The CAZymes of *S. rugosoannulata* and 14 other fungi were analyzed. To eliminate the deviation caused by different annotation pipelines, CAZymes in all fungi shown in Figure 4a were re-analyzed using the dbCAN2 database. A total of 486 CAZyme-coding genes were identified in the *S. rugosoannulata* genome, including 147 AAs, 5 CBMs, 38 CEs, 212 GHs, 69 GTs, and 15 PLs (Figure 4a, Supplementary Table S4). The *S. rugosoannulata* genome had more CAZymes than ten other fungi, although less than those in *G. marginata*, *Pleurotus ostreatus*, *Stereum hirsutum,* and *Volvariella volvacea*, indicating that *S. rugosoannulata* had high lignocellulose degradation capacity. Hierarchical clustering of the CAZyme family distribution in different species indicated that *S. rugosoannulata* was clustered with *H. sublateritium*, *P. cyanescens*, *H. marmoreus*, and *A. bisporus* (Figure 4b, Supplementary Table S4). These results were similar to those of phylogenetic analysis, indicating that evolutionarily close species have similar lignocellulose degradation profiles.

The *S. rugosoannulata* genome had more AAs (147) than all the other genomes, except for the *G. marginate* genome (155). Proteins in the AA category were mainly distributed in AA3, AA7, AA2, AA1, AA9, AA5, etc. The AA1 and AA2 categories were comprised of laccases and manganese peroxidases, respectively, two types of lignin-degrading enzymes, which was indicative of the robust lignin degradation capability of *S. rugosoannulata*. Proteins in AA3 and AA9 categories were involved in cellulose and hemicellulose degradation. GHs accounted for 43.6% of the total identified CAZymes in *S. rugosoannulata*. Twenty-five genes in GH5 and four genes in GH7 were identified in *S. rugosoannulata*, and these genes were related to cellulose digestion. Twenty-eight GH16 genes, nine GH3 genes, five GH43 genes, and four GH10 genes were also identified, and these genes were also involved in hemicellulose digestion, indicating the efficiency of this process in *S. rugosoannulata*. Proteins in the CE category were mainly distributed in CE4 and CE16 families. CE4 contained acetyl xylan esterase, which removes acetyl groups in xylan and increases the accessibility of xylan to xylanase [53]. As the main component for *S. rugosoannulata* production, maize straw is rich in xylan. Therefore, multiple copies of these genes are needed for the growth of *S. rugosoannulata* with maize straw.

### 3.5. Proteomic Analysis of Protein Expression Profiles in Different Parts of the S. rugosoannulata Fruiting Body

**Figure 5:**
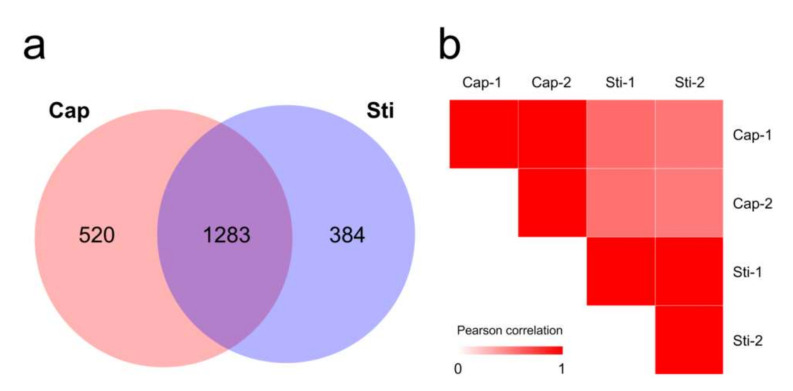
Overview of proteome data. (**a**) Venn diagram of proteins identified in the proteomes of Cap and Sti groups; (**b**) Pearson correlation coefficients for pairwise comparisons of Cap group and Sti group proteome data.

Proteomic analysis can detect protein expression profiles and generate valuable insights into complex biological processes. To evaluate the genome sequence quality and investigate the development of the *S. rugosoannulata* fruiting body, the protein expression profiles of tissues from the *S. rugosoannulata* fruiting body cap (Cap group) and stipe (Sti group) were obtained using label-free quantitative proteomic analysis. A total of 1881, 2008, 2014, and 1996 proteins were identified from four injection runs. After filtering, a total of 2187 proteins were identified (Supplementary Table S5). Among the identified proteins, 520 proteins were unique to the Cap group, and 384 proteins were unique to the Sti group (Figure 5a). Significantly different abundance protein profiles were observed when the Pearson correlation coefficient between the Cap group and the Sti group was calculated. Samples had a high correlation in the same group (~0.99) and a low correlation between the different groups (0.50–0.58) (Figure 5b). A 2.0-fold change cut-off value and a *p*-value < 0.05 were used to categorize the different abundance proteins (DAPs). Compared with the Sti group, the Cap group was associated with 216 proteins that were upregulated and 173 proteins that were downregulated (Supplementary Table S5).

**Figure 6:**
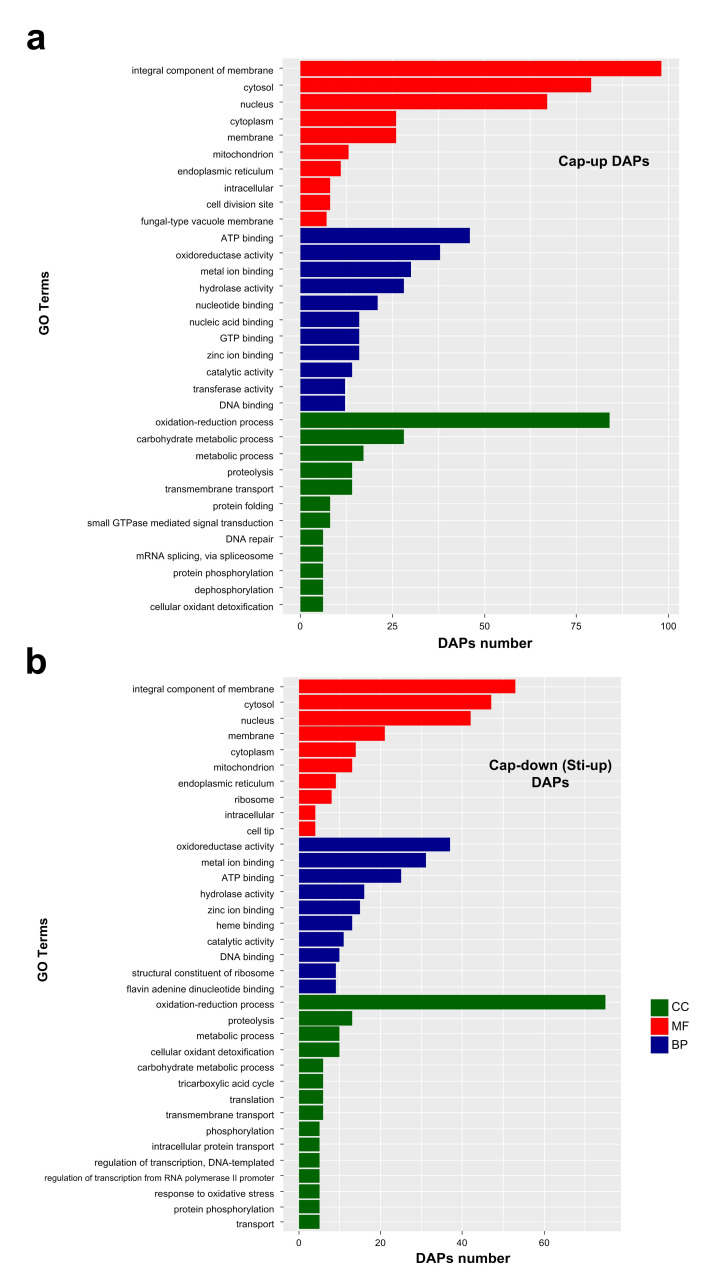
Major enriched GO terms of DAPs in molecular function (MF), cellular component (CC), and biological process (BP) categories. (**a**) Enriched GO terms of Cap-up DAPs. Cap-up DAPs, DAPs that were upregulated in the cap compared with those in the stipe; (**b**) Enriched GO terms of Cap-down DAPs. Cap-down (Sti-up) DAPs, DAPs that were downregulated in the cap compared with those in the stipe.

Most of the enriched major GO terms were the same for Cap-up and Sti-up DAPs. For the molecular function category, nucleic acid binding, GTP binding, and transferase activity were enriched as the major GO terms in the Cap-up DAPs (Figure 6a, Supplementary Table S6), whereas heme binding, structural constituents of ribosome flavin adenine and dinucleotide binding were enriched as the major GO terms in Sti-up DAPs (Figure 6b, Supplementary Table S7). For the biological process category, protein folding, small GTPase-mediated signal transduction, DNA repair, and mRNA splicing via the spliceosome were enriched as the major GO terms in Cap-up DAPs, while tricarboxylic acid cycle, phosphorylation, and response to oxidative stress were enriched as the major GO terms in Sti-up DAPs (Figure 6a,b).

**Figure 7:**
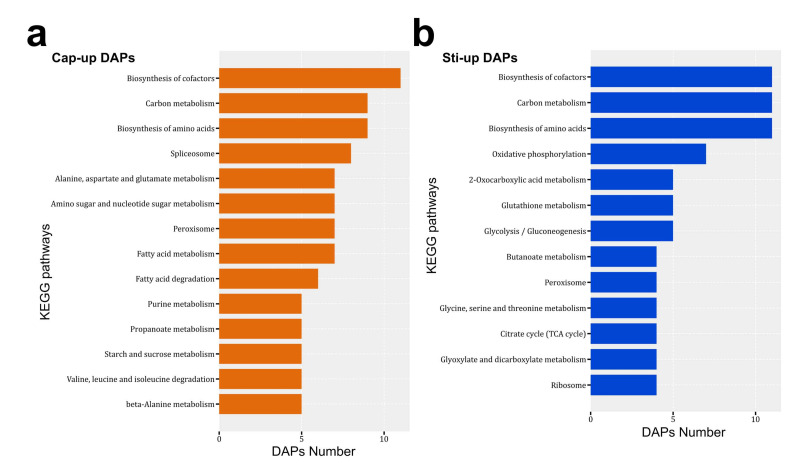
Major enriched KEGG pathways of (**a**) Cap-up DAPs and (**b**) Sti-up (Cap-down) DAPs.

Cap-up DAPs were associated with 85 KEGG pathways, and Sti-up DAPs were associated with 67 pathways (Supplementary Table S8). The biosynthesis of cofactors, carbon metabolism, biosynthesis of amino acids, and peroxisome pathways were enriched as the major pathways in both Cap-up and Sti-up DAPs (Figure 7). KEGG pathways related to the spliceosome, fatty acid metabolism, and fatty acid degradation were mainly distributed in the Cap-up DAPs (Figure 7a).

The spliceosome is a multi-megadalton ribonucleoprotein complex for pre-mRNA splicing that removes introns and forms mature RNA [54]. However, functional analysis of the spliceosome in *Basidiomycetes* is limited [55]. Recent studies have reported that the spliceosome is linked to many biological processes such as alternative mRNA splicing, development, and diseases [56,57,58]. The upregulation of spliceosome activity in the fruiting body cap compared with the stipe was not only observed in this study, but also in the proteomic analysis of the *H. marmoreus* fruiting body (in another reviewed article in JOF). The protein expression profile in cap tissues was quite different from that of stipe tissues, and meiosis was involved in the expression of specific proteins. Therefore, the demands of mRNA synthesis are higher in cap tissues than in stipe tissues. Furthermore, the upregulation of the spliceosome-related proteins indicated that there were alternative mRNA isoforms in cap tissues [57].

Fatty acid metabolism and fatty acid degradation pathways were enriched in Cap-up DAPs, indicating that fatty acid synthesis was more active in the cap than in the stipe. Previous studies have reported that the fatty acid content in the cap was higher than that in the stipe of button and beach mushrooms [59,60]. These results were consistent with those of the proteomic analysis in this study.

Proteins involved in glutathione metabolism, as well as glyoxylate and dicarboxylate metabolism, were mainly distributed in Sti-up DAPs. The Cap-up DAPs included glutathione S-transferases and catalases (MDBSrug1_05719, MDBSrug1_07056, MDBSrug1_08554, MDBSrug1_04500, and MDBSrug1_11018) and catalase (MDBSrug1_10724). Glutathione S-transferases and catalases are involved in the stress response [61]. The higher abundance of proteins involved in glutathione metabolism, as well as glyoxylate and dicarboxylate metabolism, were also observed in the stipe of *H. marmoreus* compared with the mycelia and cap (in another reviewed article in JOF). Furthermore, the upregulation of these proteins was also reported in wheat when resistance to powdery mildew was induced by sodium diethyldithiocarbamate [62] and in rhizobacteria promoted rice root [63]. Therefore, further studies are needed to analyze the functions of stress response proteins in the elongation of the fruiting body stipe.

Oxidative phosphorylation, glycolysis/gluconeogenesis, and the citrate cycle (TCA cycle) were mainly distributed in Sti-up DAPs. The expression of proteins involved in energy production and carbon metabolism indicated that stipe tissues required more carbon components and energy than cap tissues, which is consistent with the greater increase in the biomass in the stipe than in the cap. In addition, *S. rugosoannulata* obtain carbon sources by degrading lignocellulose and absorbing the degraded monosaccharides (mainly glucose) from the stipe to the cap. Therefore, the glucose concentration was higher in stipe tissues than in cap tissues. This may have also led to the higher expression of proteins involved in energy production and carbon metabolism in the stipe.

## 4. Conclusions

In summary, this is the first report of the high-quality genome of *S. rugosoannulata*. Integrity analysis and collinearity analysis revealed the high quality of the genome assembly and gene prediction. The identification of various CAZymes pointed to the efficient lignocellulose degradation capacity of *S. rugosoannulata* and explained the rapid growth of this mushroom. Proteomic analysis revealed the different metabolism processes in the cap and stipe of the *S. rugosoannulata* fruiting body, which can help us to understand the underlying mechanism of mushroom development in the future. The elucidation of the *S. rugosoannulata* genome not only provides fundamental information for the mechanical study of biological processes and agronomic traits of this mushroom, but also facilitates the genetic breeding of *S. rugosoannulata*.

## Figures and Tables

**Table 1:** De novo genome assembly and features of *S. rugosoannulata*.

Species	Strain	Sequencing Technology	Genome Size (Mbp)	GC Content (%)	Sequencing Read Coverage Depth	Contigs	Scaffold	Scaffold N50 (Mbp)
*S. rugosoannulata*	A15	Nanopore + Illumina	47.89	47.94	165.4	20	20	3.64

**Table 2:** Characteristics of the gene prediction of *S. rugosoannulata*.

Content	Number/Length
Gene number	12,752
Total concatenated gene length	26,992,228 bp (56.4% of the genome)
Average gene length	2116.71 bp
Average exon length	258.19 bp
Average exon number	6.55
Average intron length	76.75 bp

**Table 3:** Ortholog analysis of *S. rugosoannulata* and other 16 fungal species (14 *Basidiomycetes* and 2 *Ascomycetes*).

Fungal Species	Genes	Genes in Orthogroups (%)	Unassigned Genes (%)	Orthogroups Containing Species (%)	Number of Species-Specific Orthogroups	Genes in Species-Specific Orthogroups
*S.rugosoannulata*	12,752	12,342 (96.8%)	410 (3.2%)	7766 (46.6%)	197	881
*A. bisporus*	11,280	10,430 (92.5%)	850 (7.5%)	6543 (39.2%)	216	1792
*C. cinerea*	13,356	11,751 (88%)	1605 (12%)	7382 (44.3%)	371	1699
*Ganoderma lucidum*	16,495	14,002 (84.9%)	2493 (15.1%)	7274 (43.6%)	618	2796
*G. marginata*	21,391	18,671 (87.3%)	2720 (12.7%)	9196 (55.1%)	788	2960
*Hericium alpestre*	11,007	10,182 (92.5%)	825 (7.5%)	6040 (36.2%)	293	1120
*Hypsizygus marmoreus*	16,627	14,218 (85.5%)	2409 (14.5%)	8164 (48.9%)	548	2154
*H. sublateritium*	17,771	15,233 (85.7%)	2538 (14.3%)	8489 (50.9%)	466	1575
*K. phaffii*	5040	4226 (83.8%)	814 (16.2%)	3700 (22.2%)	33	92
*Lentinula edodes*	12,051	10,676 (88.6%)	1375 (11.4%)	6737 (40.4%)	415	1284
*M. conica*	11,593	7893 (68.1%)	3700 (31.9%)	5420 (32.5%)	339	1225
*P. cyanescens*	15,936	14,415 (90.5%)	1521 (9.5%)	8089 (48.5%)	395	1853
*Pleurotus ostreatus*	12,296	11,377 (92.5%)	919 (7.5%)	7233 (43.4%)	284	1077
*Stereum hirsutum*	14,066	12,588 (89.5%)	1478 (10.5%)	7510 (45%)	351	1251
*Serpula lacrymans*	14,481	12,377 (85.5%)	2104 (14.5%)	6852 (41.1%)	288	1804
*Tremella mesenterica*	8074	6264 (77.6%)	1810 (22.4%)	5079 (30.4%)	217	772
*Volvariella volvacea*	11,448	10,929 (95.5%)	519 (4.5%)	6634 (39.8%)	215	1451

## Data Availability

The raw sequence data reported in this paper have been deposited in the Genome Sequence Archive in National Genomics Data Center, China National Center for Bioinformation/Beijing Institute of Genomics, Chinese Academy of Sciences (GSA: CRA005525) that are publicly accessible at https://ngdc.cncb.ac.cn/gsa. The raw data for the proteomic analysis reported in this paper have been deposited in the OMIX, China National Center for Bioinformation/Beijing Institute of Genomics, Chinese Academy of Sciences (https://ngdc.cncb.ac.cn/omix: accession no. OMIX797).

## Supplementary Materials

The following supporting information can be downloaded at: https://www.mdpi.com/article/10.3390/jof8020099/s1, Figure S1: The position of the tissue for proteomic analysis, Figure S2: The integrated genes were derived from the statistical plots of three predictive methods, Table S1: Gene prediction of *Stropharia rugosoannulata* genome, Table S2: Sequencing results of the *S. rugosoannulata* MG69 and A15 genomes, Table S3: Annotation statistics of genomic functions of *S. rugosoannulata* using different databases, Table S4: Number of CAZymes identified in *S. rugosoannulata* and other 14 fungi, Table S5: Normalized protein abundance detected in proteomic analysis, Table S6: GO classification of upregulated DAPs in Cap group compared with Sti group, Table S7: GO classification of DAPs with lower abundance in Cap group compared with those in Sti group, Table S8: KEGG enrichment of DAPs.
