# Supplementary material for: Whole Genome Sequence of an Edible Mushroom Stropharia rugosoannulata (Daqiugaigu)
Source: J Fungi (Basel). 2022 Jan 20;8(2):99. doi: 10.3390/jof8020099 (PMC8880121; doi:10.3390/jof8020099)
Supplement: Supplementary file 1 [file jof-08-00099-s001.zip › Supplementary File(s)/Supplemental Materials.pdf]

Whole genome sequence of an edible mushroom *Stropharia rugosoannulata* (Daqiugaigu)

Shuwen Li[§1], Shuxue Zhao[§1], Chunhui Hu[1], Chengzhi Mao[1], Lizhong Guo[1], Hailong Yu[*1,2], and Hao Yu[*1]

[1]Shandong Provincial Key Laboratory of Applied Mycology, School of Life Sciences, Qingdao Agricultural University, 700 Changcheng Road,Chengyang District, Qingdao 266109, Shandong Province, People'sRepublic of China; [2]Institute of Edible Fungi, Shanghai Academy of Agricultural Sciences, National Engineering Research Center of Edible Fungi, Shanghai, 201403, China.

Running title: Genome sequence of *Stropharia rugosoannulata*

§Shuwen Li and Shuxue Zhao contributed equally to this work.

*Corresponding author:

Hao Yu E-mail: yuhaosunshine@163.com

Mailing address: 700 Changcheng Road, Chengyang District, Qingdao, Shandong Province, People's Republic of China

Hailong Yu E-mail: yuhailong@saas.sh.cn

Mailing address: 1000 Jinqi Road, Fengxian District, Shanghai, People's Republic of China

Keywords: *Genome*; *Stropharia rugosoannulata*; mushroom, lignincellulose degradation, CAZymes

Table S1. Gene prediction of *Stropharia rugosoannulata* genome. RNA-seq was used to construct the gene prediction model for gene prediction. The model was constructed using Braker software suite.

Method	Software	Species	Gene number
Ab initio based	Augustus		10,774
	Genscan		7,866
	GeneID		13,221
	GlimmerHMM		13,623
	SNAP		13,032
RNA-seq based	PASA		20,313
	TransDecoder		22,306
Homology based	GeMoMa	*Agrocybe aegerita*	8,720
		Agrocybe pediades	8,704
		Hypholoma sublateritium	11,914
Integration	EVM		12,752

Table S2. Sequencing results of the *S. rugosoannulata* MG69 and A15 genomes.

Strain	MG69	A15
Total ungapped length(bp)	50,394,922	47,892,371
Gaps between scaffolds	0	0
Genome coverage:	51x	165.4
Number of contigs	17,725	20
Contig N50	8,626	3,644,253
Contig L50(bp)	1,461	18,990
Total number of chromosomes and plasmids	0	
Number of component sequences (WGS or clone)	17,452	12,752

Table S3. Annotation statistics of genomic functions of *S. rugosoannulata* using different databases.

Database	Number (percentage)	100<=length<300	Length>=300
GO_Annotation	5,752 (45.1%)	1,357	4,295
KEGG_Annotation	3,330 (26.1%)	818	2,452
Pfam_Annotation	7,672 (60.2%)	1,820	5,751
Swissprot_Annotation	6,193 (48.6%)	1,365	4,753
Nr_Annotation	11,599 (91.0%)	3,110	8,294

Figure S1

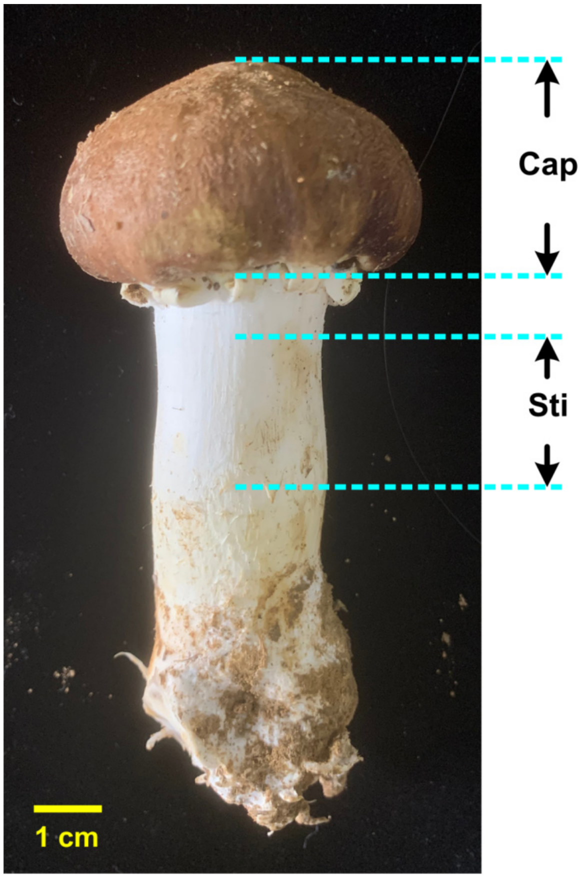

Figure S1. The position of the tissue for proteomic analysis. Cap, Cap (pileus) of the fruiting body; Sti, Stipe (stem) of the fruiting body.

Figure S2

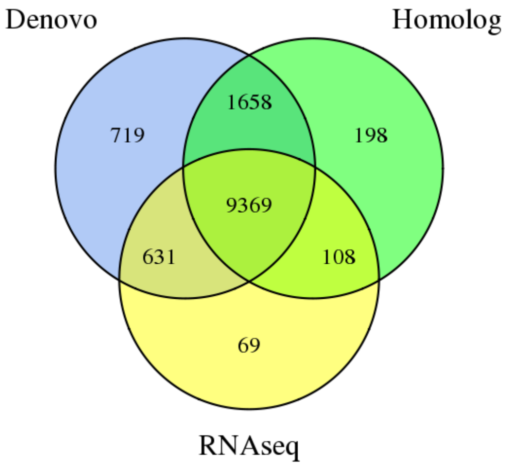

Figure S2. The integrated genes were derived from the statistical plots of three predictive method.
